# The utility of syndecan-1 circulating levels as a biomarker in patients with previous or active COVID-19: a systematic review and meta-analysis

**DOI:** 10.1186/s12879-023-08473-9

**Published:** 2023-08-04

**Authors:** Elina Ghondaghsaz, Amirmohammad Khalaji, Mitra Norouzi, Douglas D. Fraser, Sanam Alilou, Amir Hossein Behnoush

**Affiliations:** 1 Undergraduate Program in Neuroscience, University of British Columbia, Vancouver, BC Canada; 2 School of Medicine, Tehran University of Medical Sciences, Poursina St., Keshavarz Blvd, Tehran, 1417613151 Iran; 3 Faculty of Life Sciences and Biotechnology, Shahid Beheshti University, Tehran, Iran; 4 Children’s Health Research Institute, London, ON Canada; 5 Lawson Health Research Institute, London, ON Canada; 6 Department of Pediatrics, Western University, London, ON Canada; 7 Department of Physiology & Pharmacology, Western University, London, ON Canada; 8 Department of Clinical Neurological Sciences, Western University, London, ON Canada; 9 School of Medicine, Iran University of Medical Sciences, Tehran, Iran

**Keywords:** Syndecan-1, COVID-19, Diagnosis, Prognosis, Glycocalyx, Systematic review, Meta-analysis

## Abstract

**Background:**

With the emergence of coronavirus disease of 2019 (COVID-19), several blood biomarkers have been identified, including the endothelial biomarker syndecan-1, a surface proteoglycan. In the current systematic review and meta-analysis, we aimed to assess the diagnostic and prognostic role of syndecan-1 in COVID-19.

**Methods:**

PubMed, Embase, Scopus, and Web of Science, as international databases, were searched for relevant studies measuring blood syndecan-1 levels in COVID-19 patients, COVID-19 convalescents, and healthy control subjects, in patients with different COVID-19 severities and/or in COVID-19 patients with poor outcomes. Random-effect meta-analysis was performed using STATA to calculate the standardized mean difference (SMD) and 95% confidence interval (CI) for the comparison between COVID-19 patients and healthy control subjects or COVID-19 convalescents and controls.

**Results:**

After screening by title/abstract and full text, 17 studies were included in the final review. Meta-analysis of syndecan-1 levels in COVID-19 compared with healthy control subjects revealed that patients with COVID-19 had significantly higher syndecan-1 levels (SMD 1.53, 95% CI 0.66 to 2.41, *P* < 0.01). In contrast, COVID-19 convalescent patients did not show significant difference with non-convalescents (SMD 0.08, 95% CI -0.63 to 0.78, *P* = 0.83). Regarding disease severity, two studies reported that more severe forms of the disease were associated with increased syndecan-1 levels. Moreover, patients who died from COVID-19 had higher syndecan-1 levels compared with survivors (SMD 1.22, 95% CI 0.10 to 2.33, *P* = 0.03).

**Conclusion:**

Circulating syndecan-1 level can be used as a biomarker of endothelial dysfunction in COVID-19, as it was increased in COVID-19 patients and was higher in more severe instances of the disease. Further larger studies are needed to confirm these findings and further enlighten the role of syndecan-1 in clinical settings.

**Supplementary Information:**

The online version contains supplementary material available at 10.1186/s12879-023-08473-9.

## Introduction

Coronavirus disease 2019, known as COVID-19 and caused by severe acute respiratory syndrome coronavirus 2 (SARS-COV-2), is a multisystem disease mainly causing respiratory symptoms [1]. Besides reverse transcriptase polymerase chain reaction (RT-PCR) as the mostly-used diagnostic tool currently, several biomarkers have been introduced for the diagnosis and prognosis of the disease. Hence, novel biomarkers able to differentiate COVID-19 cases from healthy control subjects might be beneficial in clinical settings.

There is evidence for endothelial dysfunction in the pathogenesis of COVID-19 in both direct and indirect ways [2]. It has been suggested that COVID-19 infection leads to several endothelial-related phenomena which include, but are not limited to, reduced nitric oxide (NO) bioavailability, oxidative stress, endothelial toxicity, and glycocalyx/barrier disruption [2–4]. Moreover, severe COVID-19 is more common in patients with comorbidities such as cardiovascular and renal diseases, mostly with endothelial dysfunction [5].

Syndecan is a member of the surface proteoglycans family which carries glycosaminoglycan chains of heparan sulfate or chondroitin sulfate. It consists of four different types, encoded by different genes, among which syndecan-1 has been assessed in different diseases [6, 7]. During illness, syndecan-1 is degraded by several matrix metalloproteinases and ADAM17 [7] and may be a biomarker candidate for the diagnosis and prognosis of COVID-19. In fact, numerous studies have investigated syndecan-1 levels in hospitalized COVID-19 patients, as well as in COVID-19 convalescence.

In this systematic review and meta-analysis, we present the findings from studies that reported syndecan-1 levels in COVID-19 patients or convalescents and compared them with controls. Also, we reviewed the possible changes in syndecan-1 levels with regard to COVID-19 complications such as ICU admission and death. The findings of the current study can guide researchers in future investigations of this biomarker.

## Methods

### Search strategy

Our study was performed in accordance with the Preferred Reporting Items for Systematic Reviews and Meta-Analysis (PRISMA-2020) guidelines [8]. Search terms related to “syndecan-1” and “COVID-19” were used for search in PubMed, Scopus, Embase, and Web of Science until December 2022. A manual review of the references list of included studies was also performed to find any possible missed studies. Details of the search strategy in each database are shown in Supplementary Table 1.

### Inclusion criteria, screening, and data extraction

Studies were included if they have reported syndecan-1 levels in serum/plasma of COVID-19 patients or convalescents and compared them with healthy control subjects or if they assessed syndecan-1 levels within different stages of COVID-19 or complications associated with the disease. We included case-control, cross-sectional, retrospective cohort, and prospective cohort studies while case reports, case series, congress abstracts, and reviews were excluded. Our research question in PECO format (population, exposure, comparison, and outcome) is as follows: P) patients with a confirmed diagnosis of COVID-19 and/or COVID-19 convalescents, E) previous or active SARS-CoV-2 infection, C) healthy controls or different severities of COVID-19, and O) diagnostic, prognostic, and discriminatory ability of syndecan-1 in these patients.

Two authors independently screened the studies first by title and abstract and then with full text. Any case of disparities was resolved by discussion with a third author (EG). Then, data were extracted independently by two authors (AK and AHB). The data extracted included the followings: (1) first author’s name, (2) publication year, (3) country in which the study was performed, (4) the population assessed, (5) mean age of participants, (6) male percentage in the study population, (7) main findings of each study in relation to syndecan-1, (8) diagnostic measures of syndecan-1 in patients with COVID-19 (area under the receiver operating characteristics curve (AUC-ROC) in addition to sensitivity and specificity), and (9) syndecan-1 levels in each of the study’s groups.

### Quality assessment

The qualities of included studies were assessed using the Newcastle Ottawa Scale (NOS) for non-randomized studies designed for the determination of the risk of bias [9]. This system includes three domains of selection, comparability, and outcome as the potential sources of bias. The overall quality of each study is categorized as “very good,” “good,” “satisfactory,” or “unsatisfactory” based on the scores of 9–10, 7–8, 5–6, and < 5, respectively. Two independent authors (MN and EG) assessed the qualities and discussed them with a third author (AHB) in case of disagreement.

### Statistical analysis

Meta-analysis was performed with STATA (version 17, Stata Corp.) to calculate the standardized mean difference (SMD) in addition to a 95% confidence interval (CI) for assessment of the difference between the two groups (COVID-19 vs. controls or convalescent COVID-19 vs. controls). A *P* of < 0.05 was considered statistically significant. Due to high heterogeneity among studies, the random-effect model was used (restricted maximum likelihood (REML)). A *P* of < 0.05 was considered a statistically significant result.

As some of the studies reported syndecan-1 levels as the median and interquartile range (IQR), we used the methods suggested by Luo et al. [10] and Wan et al. [11] to convert them to mean and standard deviation (SD) in order to perform the meta-analysis. The heterogeneity was calculated with Cochrane’s Q and Higgin’s I^2^ test with thresholds of ≤ 25% for low, 26–75% for moderate, and > 75% for high [12]. Subgroup analysis was performed, when possible, to investigate the effect of disease severity on the pooled effect size. Meta-regression was performed for the association of SMD with publication year, sample size, mean age, and male percentage of the COVID-19 group in each of the studies. The bubble plots were designed to show these analyses as well. Finally, publication bias was assessed by visual inspection of funnel plots and Egger’s [13] and Begg’s [14] statistical tests. Finally, a random-effect meta-analysis was performed for AUCs of syndecan-1 for mortality prediction, obtained by the studies.

## Results

### Search results and study characteristics

**Fig. 1:**
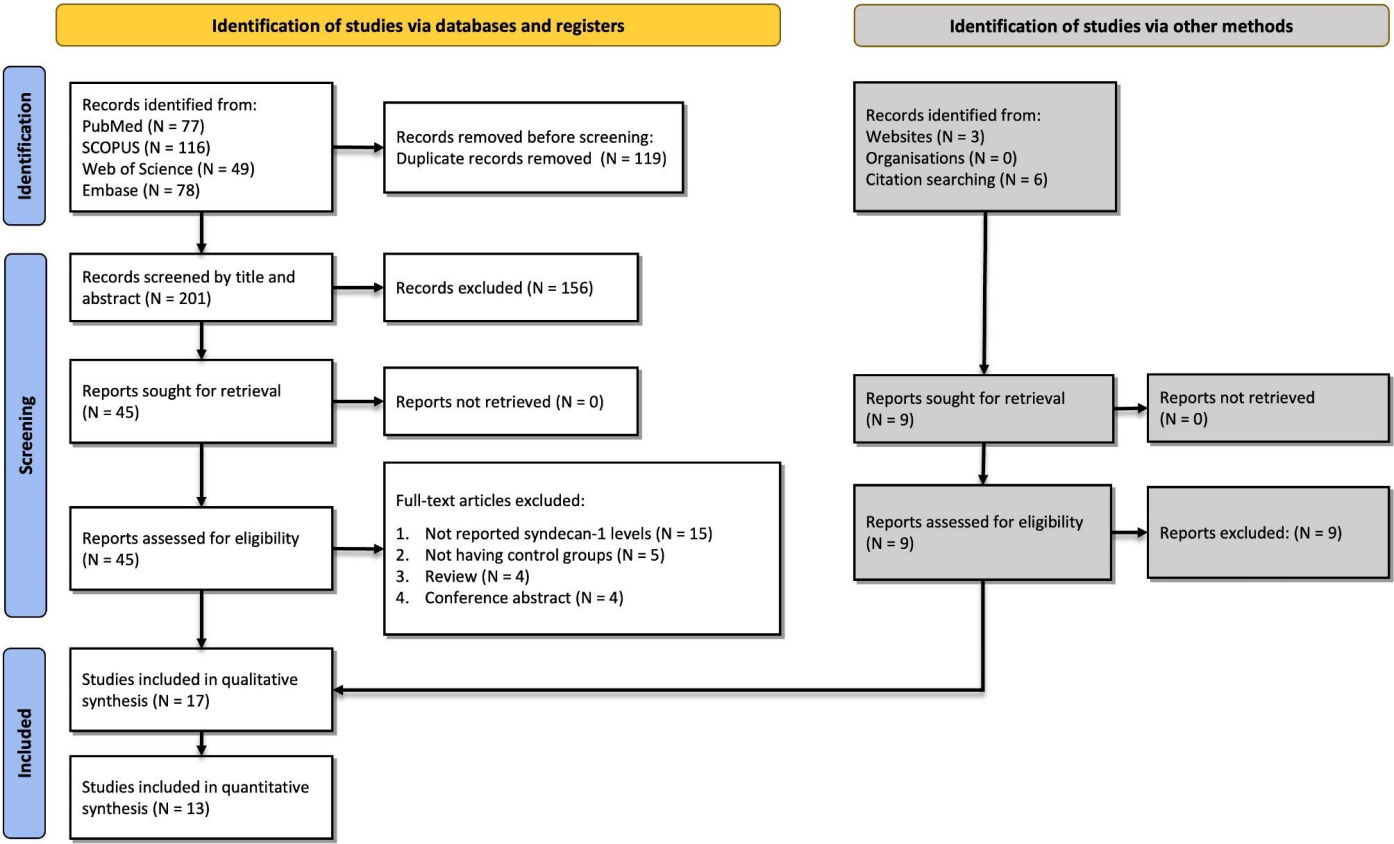
Flow diagram summarizing the selection of eligible studies based on the PRISMA guidelines

Our search resulted in a total of 320 records while 119 were duplicates. From 201 records undergoing title and abstract assessment, 156 were excluded and after evaluating those with full texts, 17 studies were included in our review [15–31], of which meta-analysis was performed in 13 studies. A detailed PRISMA diagram showing the selection process of studies is available in Fig. 1.

Eleven studies compared syndecan-1 levels between patients with COVID-19 and healthy control subjects [15–25], three studies compared syndecan-1 levels between COVID-19 convalescents and healthy control subjects [24, 26, 27], while eight studies evaluate the association between syndecan-1 levels and complications of COVID-19 [19, 22, 23, 25, 28–31]. The diagnosis of COVID-19 was confirmed by RT-PCR test in all the included studies. Study characteristics of all included studies are available in Tables 1 and 2. Moreover, all included studies were classified as either “good” or “very good” based on NOS criteria (Table 3).

**Table 1:** Baseline characteristics of the studies assessing syndecan-1 in COVID-19 vs. control

Study	Year	Country	Population	N (COVID/Control) or (Convalescent/control)	Age (Year)	Male sex (%)	Main findings
**COVID-19 vs. Control**							
Astapenko et al.	2022	Czech Republic	Adult patients with septic shock associated with COVID-19 pneumonia or bacterial pneumonia (control)	28 (15/13)	67.0 ± 9.6	67.8	Syndecan-1 was significantly different between the groups over the monitored days of the ICU stay (ANOVA *P* < 0.0001)
Fraser et al.	2020	Canada	COVID-19 patients admitted to ICU and age- and sex-matched healthy controls	20 (10/10)	59.3 ± 9.6	30.0	Syndecan-1 levels were significantly higher in COVID-19 patients compared to healthy controls (181.9 [103.6-313.3] ng/mL vs. 76.0 [26.3–97.6] ng/mL; *P* = 0.004)
Goonewardena et al.	2021	United States	Hospitalized symptomatic COVID-19 patients and healthy controls	19 (12/7)	54.4 ± 8.3	100	Syndecan-1 levels were significantly higher in COVID-19 patients compared to healthy controles (247.37 [101.43–458.26] ng/ml vs. 84.8 [52.88–123.59] ng/ml; *P* = 0.036)
Hutchings et al.	2021	United Kingdom	Patients with confirmed positive PCR for SARS-COV-2 infection treated in one of the intensive care units of King’s College Hospital and healthy controls	42 (30/12)	49.9 ± 9.5	64.3	COVID-19 patients had significantly higher levels of syndecan-1 compared to healthy controls (*P* = 0.04)
Karampoor et al.	2021	Iran	COVID-19 patients (hospitalized in ICU and moderate severity group) and healthy controls	176 (120/56)	56 [IQR 39–66]	57.9	Patients with COVID-19 had significantly higher levels of syndecan-1 compared to healthy controls (*P* < 0.001). Moreover, syndecan-1 levels were higher in COVID-19 patients admitted to ICU compared to non-ICU ones (*P* < 0.001)
Kim et al.	2021	South Korea	Severe COVID-19 patients and healthy controls	44 (31/13)	NR	NR	Syndecan-1 levels were significantly higher in COVID-19 patients compared to healthy controls (5401 [4208–7734] pg/ml vs. 3091 [2572–3233] pg/ml; *P* < 0.001)
Maldonado et al.	2022	Chile	Patients aged 18 years and older with clinically suspected and laboratory (RT-PCR)-confirmed SARS-CoV-2 infection, hospitalized in critical patient care units with the need for HFNO or mechanical ventilation during the first 24 h after arriving at the institute, and volunteers without COVID-19 (healthy control)	52 (43/9)	NR	NR	Syndecan-1 levels were significantly higher in COVID-19 patients after 24 h and 10 days, compared with healthy controls (*P* < 0.05 and *P* < 0.0001, respectively)
Mobayen et al.	2021	United Kingdom	Patients receiving hemodialysis with positive COVID-19 RT-PCR and hemodialysis patients without clinical signs and laboratory serological signs of COVID-19 (controls)	49 (39/10)	Range 23–86	73.5	Patients with severe COVID-19 had significantly higher syndecan-1 levels in comparison with controls (*P* < 0.005)
Rovas et al.	2021	Germany	Hospitalized adult patients with moderate-to-severe or critical COVID-19	38 (23/15)	NR	NR	COVID-19 patients with mechanical ventilation had higher syndecan-1 levels compared with healthy controls (*P* < 0.0001) while this was not significant for comparison of COVID-19 cases without need for mechanical ventilation with healthy controls (*P* = 0.09)
Vollenberg et al.	2021	Germany	Hospitalized COVID-19 patients and preexisting conditions-free age-matched healthy controls	24 (10/14)	55.7 ± 6.3	60.9	Syndecan-1 levels were significantly higher in hospitalized COVID-19 patients compared to age-matched healthy controls (142 [85.2-181.3] ng/ml vs. 31.6 [17.1–54.7] ng/ml; *P* ≤ 0.01)
Yuan et al.	2022	Netherlands	Hospitalized patients with positive PCR test and age-matched healthy controls	44 (32/12)	NR	NR	There was no significant difference between COVID-19 patients and healthy controls in terms of syndecan-1 levels (*P* > 0.05)
**Convalescent vs. Control**							
Hetland et al.	2022	Norway	COVID-19 convalescent blood donors (mostly positive RT-PCR test), and healthy employees with a negative RT-PCR test	156 (129/27)	NR	NR	Syndecan-1 was not statistically different between convalescents and healthy controls (*P* = 0.137)
Kozlowski et al.	2022	Poland	Volunteer blood donors of which some had mild-to-moderate previous SARS-CoV-2 infection at least 6 months before blood donation.	294 (215/79)	Range 18–65	NR	Lower levels of syndecan-1 was found in convalescents in comparison to those of the control group (*P* = 0.0082)
Vollenberg et al.	2021	Germany	SARS-CoV-2-infected (RT-PCR positive) individuals with mild disease course (no inpatient treatment) who recovered from infection in the outpatient clinic and were healthy without no known pre-existing conditions or medications (convalescent group). Age-matched subjects without any pre-existing conditions were healthy controls.	38 (24/14)	55.5 ± 3.5	70.3	The convalescent group after a mild disease had significantly elevated syndecan-1 levels compared to the healthy population (*P* < 0.05)

Data are presented as mean ± standard deviation, median (range), or median [interquartile range]. COVID-19: coronavirus disease 2019, SARS-CoV-2: severe acute respiratory syndrome coronavirus 2, ICU: intensive care unit, PCR: polymerase chain reaction, ANOVA: analysis of variance, IQR: interquartile range, HFNO: high-flow nasal oxygen

**Table 2:** Baseline characteristics of the studies assessing syndecan-1 in COVID-19 complications

Study	Year	Country	Population	N total COVID-19	Complication or severity	Main findings
Mobayen et al.	2021	United Kingdom	Patients receiving hemodialysis and had a positive RT-PCR test result (COVID-19) that were further categorized into mild (outpatient) or severe hospitalized cases	39	Severity (mild and moderate)	Patients with severe COVID-19 had significantly higher syndecan-1 levels, compared with mild cases (*P* < 0.05)
Ogawa et al.	2021	Japan	COVID-19 patients (except for those without symptoms or mild symptoms) undergoing standard treatment, including intensive care at the hospital	15	Severity (severe and critical)	Syndecan-1 levels were elevated in critical COVID-19 cases compared with severe COVID-19 patients, both at admission and over the time of hospitalization (*P* < 0.05)
Karampoor et al.	2021	Iran	COVID-19 patients (hospitalized in ICU and moderate group)	120	ICU admission and death	Syndecan-1 levels were higher in ICU patients (*N* = 63), compared to non-ICU ones (*N* = 57) (*P* < 0.001) Also, patients who died from COVID-19 had significantly higher syndecan-1 levels than those alive (*P* < 0.001)
Yuan et al.	2022	Netherlands	Hospitalized patients with positive RT-PCR tests	32	ICU admission	There was no difference between ICU and non-ICU COVID-19 patients in terms of syndecan-1 levels (*P* > 0.05)
Zhang et al.	2021	China	Hospitalized adult patients diagnosed with COVID-19 (RT-PCR-confirmed) who were admitted to ICU	49	Death	Survivors had significantly lower levels of syndecan-1 levels (median 504.0 ng/mL vs. 1031.4 ng/mL, *P* = 0.002)
Dupont et al.	2021	France	COVID-19 patients admitted to ICU	82	High-flow oxygen therapy, respiratory failure, liver injury, multi-organ injury, and death	Baseline syndecan-1 levels were significantly higher in patients requiring high-flow oxygen therapy or mechanical ventilation compared with cases with lower oxygen requirements (*P* < 0.0001) Moreover, syndecan-1 was significantly associated with respiratory failure at admission or during follow-up, liver injury, and multi-organ failure.
Kweon et al.	2021	South Korea	Severe hospitalized COVID-19 patients receiving oxygen therapy	56	Weaning failure (death or discharge with oxygen device before day 28 of admission)	Syndecan-1 was higher in the weaning failure group, compared with the weaning success which was not significant 9000 [5581–12,353] pg/ml vs. 5969 [4734–7670] pg/m, *P* = 0.06)
Rovas et al.`	2021	Germany	Hospitalized adult patients with moderate-to-severe or critical COVID-19	23	Mechanical ventilation	Syndecan-1 was significantly higher in patients with mechanical ventilation (239.0 [162.8–251.5] ng/ml vs. 29.9 [22.8–82.4] ng/ml, *P* < 0.0001)

Data are presented as median [interquartile range]. COVID-19: coronavirus disease of 2019, RT-PCR: reverse transcription-polymerase chain reaction, ICU: intensive care unit

**Table 3:** Quality Assessment of Included Studies Based on the Newcastle-Ottawa Scale (NOS)

Study	Selection				Comparability	Outcome		Overall Score
	Representation	Sample size	Non-Respondents	Exposure		Outcome	Statistical test	
Astapenko et al. (2022)	*	*	*	**	-	**	*	8
Fraser et al. (2020)	*	*	*	**	**	**	*	10
Dupont et al. (2021)	*	*	*	**	-	**	*	8
Goonewardena et al. (2021)	*	*	*	**	-	**	*	8
Hetland et al. (2022)	*	*	*	**	-	**	*	8
Hutchings et al. (2021)	*	*	*	**	-	**	*	8
Karampoor et al. (2021)	*	*	*	**	-	**	*	8
Kim et al. (2021)	*	*	*	**	**	**	*	10
Kozlowski et al. (2022)	*	*	*	**	-	**	*	8
Kweon et al. (2021)	*	*	*	**	-	**	*	8
Maldonado et al. (2022)	*	*	*	**	-	**	*	8
Mobayen et al. (2021)	*	*	*	**	-	**	*	8
Ogawa et al.	*	*	*	**	**	**	*	10
Rovas et al. (2021)	*	*	*	**	-	**	*	8
Vollenberg et al. (2021)	*	*	*	**	**	**	*	10
Yuan et al. (2022)	*	*	*	**	**	**	*	10
Zhang et al. (2021)	*	*	*	**	-	**	*	8

### Syndecan-1 levels between patients with COVID-19 vs. healthy control subjects

Eleven studies evaluated syndecan-1 levels in patients with COVID-19 and healthy individuals [15–25]. All studies have been published between 2020 and 2022. The baseline characteristics of these studies are available in Table 1. Although the study by Yuan et al. [25] found no difference between syndecan-1 levels in patients with COVID-19 compared to controls, other 10 studies found significantly higher levels of syndecan-1 in COVID-19 patients in comparison to healthy individuals [15–24].

#### Meta-analysis

**Fig. 2:**
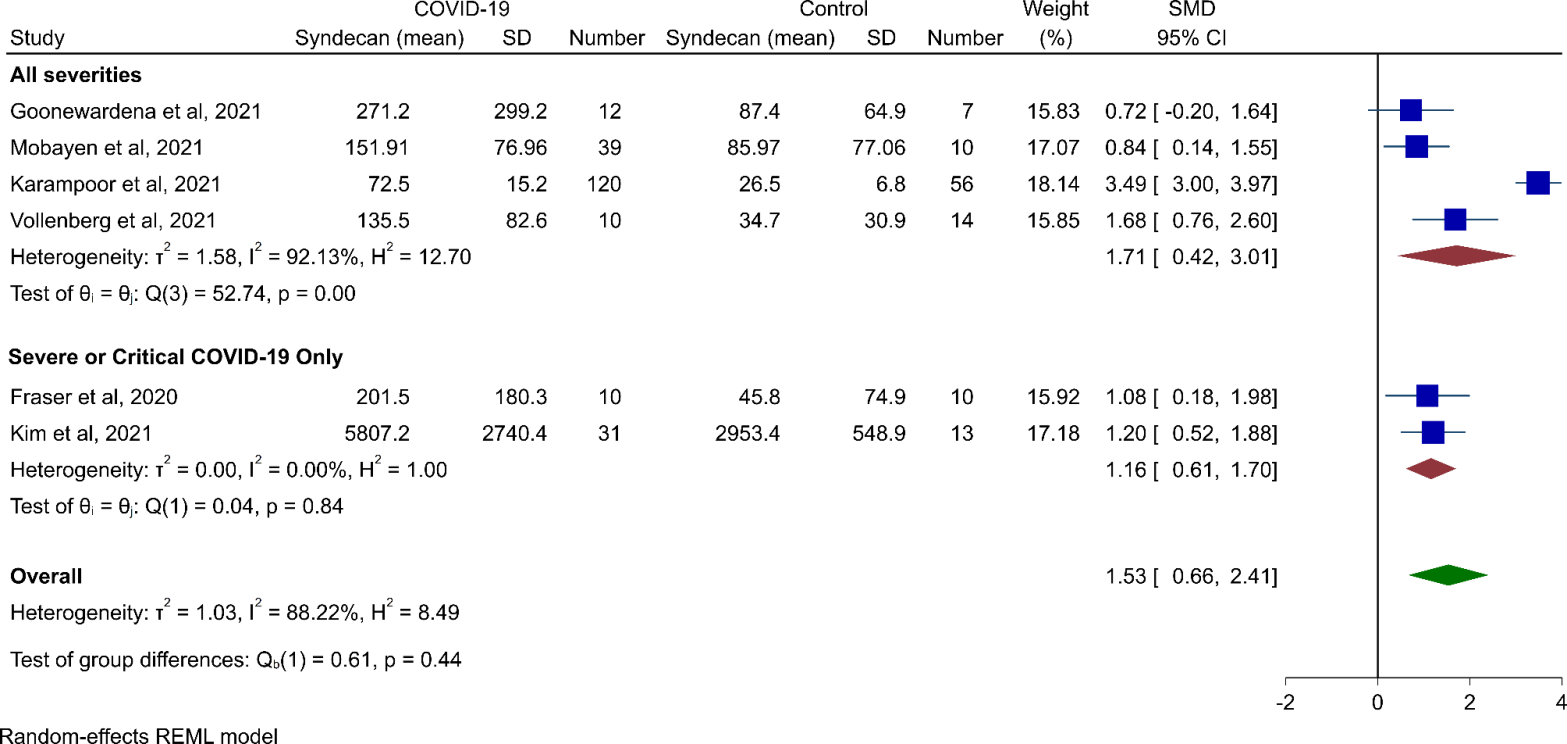
Meta-analysis results and subgroup analysis for comparison syndecan-1 levels between COVID-19 patients and healthy control subjects

Figure 2 illustrates a meta-analysis comparing syndecan-1 levels between active SARS-CoV-2 infection and healthy control subjects. In this meta-analysis of six studies [16, 17, 19, 20, 22, 24], we found significantly higher syndecan-1 concentration in patients with COVID-19 compared to healthy control subjects (SMD 1.53, 95% CI 0.66 to 2.41, p-value < 0.01). However, the heterogeneity was high (*I*^*2*^ = 88.22%). Subgroup analysis based on severity showed that in both groups, severe/critical only and all severities, there were higher levels of syndecan-1 in patients compared to controls (all severities: SMD 1.71, 95% CI 0.42 to 3.01, *P* < 0.01; severe/critical only: SMD 1.16, 95% CI 0.61 to 1.70, *P* < 0.01). Finally, as the study by Mobayen et al. (2021) [22] investigated the population undergoing hemodialysis, we performed the meta-analysis without this study to investigate its impact on the pooled effect size. As shown in Supplementary Fig. 1, the result remained significant (SMD 1.67, 95% CI 0.67 to 2.68, *P* < 0.01).

#### Publication bias

The funnel plot assessing publication bias in comparing syndecan-1 levels between COVID-19 patients and controls is available in Supplementary Fig. 2. It shows asymmetry suggesting two missing studies and the possibility of publication bias. In line with the funnel plot, Egger’s statistical test showed significant publication bias in this meta-analysis (*P* = 0.035). However, Begg’s test found no significant publication bias (*P* = 0.707).

#### Meta-regression

Meta-regression found that mean age and sample size had a significant association with the previously mentioned results (*P* = 0.009 and *P* < 0.001, respectively). R^2^ analog was 60.39% for mean age and 86.21% for sample size. Meta-regression with publication year and male sex percentage revealed no significant association with the results (*P* = 0.683 and *P* = 0.632, respectively). Table 4 explains the meta-regression of syndecan-1 in patients with COVID-19 compared to healthy control subjects. Supplementary Figs. 3–6 show bubble plots for meta-regression based on mean age, publication year, male sex percentage, and sample size, respectively.

**Table 4:** Meta-regression of syndecan-1 levels in patients with COVID-19 vs. controls

Moderator	No. of Comparisons		Meta-regression				R^2^ Analog (proportion of variance explained)
	COVID-19	Control	Slope	95% confidence interval		*P*	
**Mean Age (years)**	222	110	-0.139	-0.244	-0.035	0.009	60.39%
**Publication Year**	222	110	0.536	-2.033	3.104	0.683	0%
**Male sex (percentage)**	222	110	-0.009	-0.046	0.028	0.632	0%
**Sample Size**	222	110	0.022	0.013	0.032	< 0.001	86.21%

### Syndecan-1 levels between different severities of COVID-19

Two studies compared syndecan-1 levels between different severities of the COVID-19 [22, 28]. Mobayen et al. [22] compared the patients with mild COVID-19 (defined as remaining outpatient for the duration of infection) and severe COVID-19 (according to the World Health Organization criteria for severe disease: respiratory rate ≥ 30/min, blood oxygen saturation ≤ 90%, arterial oxygen partial pressure: fractional inspired oxygen ratio < 300, or infiltrates affecting 50% of the lung field within 24–48 h). They found higher plasma syndecan-1 levels in patients with severe COVID-19 (148.5 [103.3–203.3] ng/ml) compared to mild COVID-19 cases (63.8 [49.0–138.6] ng/ml; *P* < 0.05) and healthy control subjects (48.0 [44.9–73.3] ng/ml; *P* < 0.005). Moreover, Ogawa et al. [28] investigated the difference between severe COVID-19 (defined as SpO2 ≤ 94%, requiring oxygen support) and critical COVID-19 (defined as requiring heart-lung machine or extracorporeal membrane oxygenation (ECMO) support for acute respiratory distress syndrome (ARDS)). They found higher syndecan-1 levels in critical COVID-19 patients compared to severe COVID-19 cases (*P* < 0.05).

### Meta-analysis of syndecan-1 levels between COVID-19 convalescents vs. healthy control subjects

**Fig. 3:**
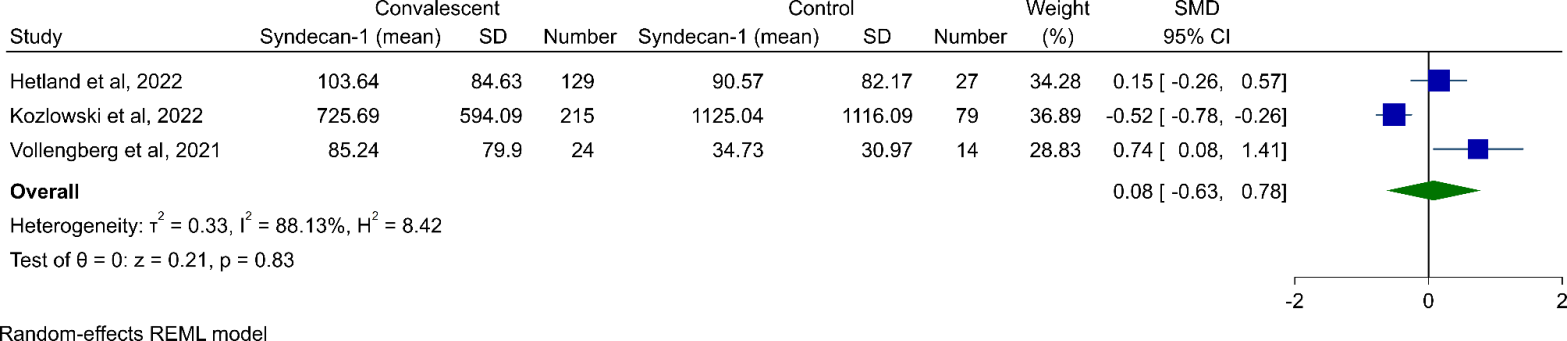
Meta-analysis results for comparison syndecan-1 levels between convalescent COVID-19 individuals and controls

The baseline characteristics of three studies that compared syndecan-1 levels between COVID-19 convalescents and healthy participants [24, 26, 27] are available in Table 1. We performed a meta-analysis of syndecan-1 levels between COVID-19 convalescents and patients without prior COVID-19 infection (Fig. 3). In a pooled meta-analysis of three studies, we found no significant difference in syndecan-1 levels between COVID-19 convalescents and healthy control subjects (SMD 0.08, 95% CI -0.63 to 0.78, *P* = 0.83) associated with high heterogeneity (*I*^*2*^ = 88.13%).

### Syndecan-1 as a prognostic factor in patients with COVID-19

Six studies evaluated the prognostic role of syndecan-1 in patients with COVID-19 [19, 23, 25, 29–31]. Table 2 describes the baseline characteristics and main findings of these studies. The association between syndecan-1 levels and mortality [19, 29], ICU admission [19, 25], and the need for mechanical ventilation [23, 30, 31] were assessed in these studies.

#### Mortality

Three studies compared syndecan-1 levels between alive and dead patients with COVID-19 [19, 29, 30]. Dupont et al. [30] reported higher levels of syndecan-1 in dead patients with COVID-19, compared to survivors, although not significant (239 [122–505] vs. 142 [82–297] ng/ml). Karampoor et al. [19] found that syndecan-1 levels were significantly higher in patients who died compared to alive ones (116 [85–127] ng/ml vs. 71 [62–79] ng/ml; *P* < 0.001). In line with the previous study, a study by Zhang et al. [29] found higher levels of syndecan-1 in non-survivors compared to alive patients (1031.4 ng/ml vs. 504.0 ng/ml; *P* = 0.002). A cut-off of 813.8 ng/ml for syndecan-1 can distinguish survivors from non-survivors with an area under the curve of 0.783 [95% CI 0.647–0.918; *P* = 0.002], the sensitivity of 0.686 and specificity of 0.786.

**Fig. 4:**
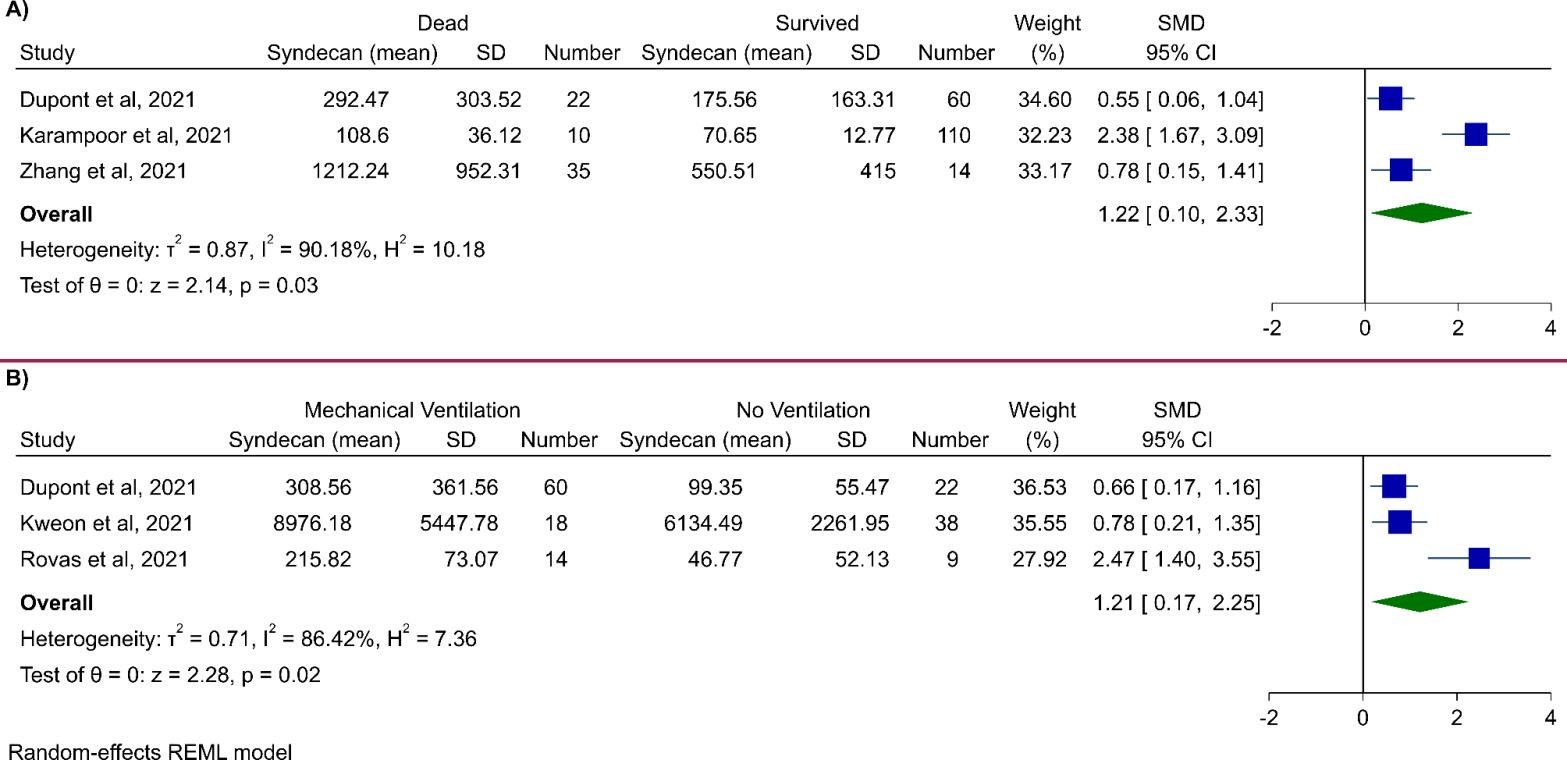
Meta-analysis of syndecan-1 levels for comparison of **(A)** dead and survived patients, and **(B)** patients with and without need for mechanical ventilation

Meta-analysis of these studies revealed that patients who died from COVID-19 had significantly higher levels of syndecan-1, compared with those who survived (SMD 1.22, 95% CI 0.10 to 2.33, *P* = 0.03). This analysis was associated with a high level of heterogeneity (*I*^*2*^: 90.2%). The forest plot for this meta-analysis is illustrated in Fig. 4A.

#### ICU admission

Studies by Karampoor et al. [19] and Yuan et al. [25] compared syndecan-1 levels between ICU and non-ICU patients. Karampoor et al. found significantly higher syndecan-1 levels in ICU patients compared to non-ICU ones (76 [69–87] ng/ml vs. 67 [56–78] ng/ml; *P* < 0.001). However, Yuan et al. [25] found no significant difference between ICU and non-ICU patients with COVID-19 (*P* > 0.05).

#### Need for mechanical ventilation

Three studies evaluated the association between syndecan-1 levels and the need for mechanical ventilation [23, 30, 31]. Dupont et al. [30] found that levels of syndecan-1 were significantly higher in patients receiving high-flow oxygen therapy or mechanical ventilation compared to other COVID-19 patients (*P* < 0.001). Kweon et al. [31] found that patients with weaning (liberation from oxygen therapy) failure had marginally insignificant higher syndecan-1 levels compared to patients with successful weaning (9000 [5581–12,353] pg/ml vs. 5969 [4734–7670] pg/ml; *P* = 0.06). Finally, Rovas et al. [23] found that patients who underwent mechanical ventilation had significantly higher syndecan-1 levels compared to COVID-19 patients without the need for mechanical ventilation (*P* < 0.001).

Meta-analysis was performed for comparison of syndecan-1 levels in COVID-19 patients with and without the need for mechanical ventilation. It was found that patients with a need for mechanical ventilation had higher levels of syndecan-1 (SMD 1.21, 95% CI 0.17 to 2.25, *P* = 0.02, Fig. 4B).

### Diagnostic and prognostic measures of syndecan-1 in COVID-19

The AUC-ROC analysis of syndecan-1 levels was performed in four studies [19, 21, 23, 29]. Karampoor et al. [19] reported an AUC of 0.705 for syndecan-1 in the prediction of ICU admission in patients hospitalized with COVID-19. Similarly, the study by Rovas et al. [23] reported AUCs of 0.91 and 0.76 for this biomarker in the prediction of the development of moderate-to-severe ARDS and thrombotic events. In terms of COVID-19 mortality, this study reported an AUC of 0.65 (95% CI 0.42 to 0.87). Maldonado et al. [21] found specificity and sensitivity of 100% and 81.82% for the prediction of COVID-19 mortality with a cut-off value of 40.1 ng/ml while AUC was 0.94 (95% CI 0.84 to 1.00). In a similar analysis by Zhang et al. [29], the AUC of syndecan-1 was identified as 0.783 (95% CI 0.647 to 0.918) with 68.6% sensitivity and 78.6% specificity (cut-off 813.8 ng/ml). The pooled effect size for AUCs of studies predicting mortality is illustrated in Supplementary Fig. 7. The overall AUC of syndecan-1 in the prediction of mortality was calculated as 0.81 (95% CI 0.65 to 0.98).

## Discussion

In this systematic review and meta-analysis, we found higher levels of syndecan-1 in patients with COVID-19 compared to healthy control subjects through meta-analysis, in addition to higher syndecan-1 levels in higher severities of COVID-19 based on two individual studies. In a separate analysis, there was no difference in syndecan-1 levels between COVID-19 convalescents and individuals without prior SARS-CoV-2 infection. Finally, we reviewed the potential prognostic role of syndecan-1 in these patients for mortality, ICU admission, and the need for mechanical ventilation. Higher syndecan-1 levels were shown in patients who died from COVID-19 and those with the need for mechanical ventilation.

Syndecan-1 and endocan are two main markers of glycocalyx damage [32], used to detect endothelial dysfunction in serum or plasma. Several studies compared the usefulness of endocan compared to syndecan-1 as diagnostic or prognostic biomarkers; however, a study conducted by Smart et al. found that syndecan-1 is a stronger predictor of respiratory failure, compared to endocan [33]. Moreover, a scoping review by Yanase et al. [34] found that syndecan-1 was the most frequently reported marker of glycocalyx damage in the healthy population, emphasizing the importance of this marker compared to other glycocalyx biomarkers (e.g., endocan, heparan sulfate, or hyaluronic acid). All in all, syndecan-1 is a useful biomarker of glycocalyx damage and can be used to detect endothelial dysfunction in several populations including COVID-19 patients [7].

The role of inflammatory biomarkers in COVID-19 has been shown previously [35–38]. Syndecans are inflammatory biomarkers that regulate cytokine function and leukocyte extravasation [39]. Moreover, the expression levels of syndecans can change during inflammation due to cytokine-mediated changes. Hayashida et al. [40] found that syndecan-1 shedding is essential in the resolution of inflammatory processes in mice by removing sequestered CXC chemokines including macrophage inflammatory protein-2 and keratinocyte cytokine. In addition, a study by Zhang et al. [41] found that syndecan-1 rescues acute lung injury via a signaling axis mainly by mitigating the expression of pro-inflammatory cytokines. Several studies have evaluated the role of syndecan-1 in infections, such as herpes simplex virus, human immunodeficiency virus, and staphylococcus aureus [42–45]. With the emergence of COVID-19, studies evaluated the role of syndecan-1 as a biomarker in distinguishing COVID-19 patients from controls, the severity of COVID-19, and its role as a prognostic marker.

To the best of our knowledge, this is the first meta-analysis comparing syndecan-1 levels between COVID-19 patients, convalescents, and healthy control subjects. Although lab tests are not routinely used in the work-up of patients exposed to COVID-19 infection, syndecan-1 could act as a promising biomarker of COVID-19 infection in exposed individuals for the following reasons. First, in line with our results, almost all studies comparing syndecan-1 levels between COVID-19 cases and healthy control subjects found higher levels in COVID-19 patients compared to healthy individuals. Especially, based on our meta-regression results for the mean age of patients, younger ages of COVID-19 patients were associated with larger levels of difference, emphasizing its importance in this vulnerable group. Second, this marker is not elevated in convalescent COVID-19 patients, making this biomarker unique and specific since it is only increased in the active form of the disease. Finally, measuring syndecan-1 levels showed promising results in predicting adverse in-hospital and short-term events including the need for ICU admission, the need for mechanical ventilation, and death.

Regarding convalescents, there were controversies in the studies, so, among the three studies we used in the meta-analysis, one study reported significantly higher syndecan-1 levels while the other reported significantly lower syndecan-1 levels in patients with COVID-19. Interestingly, in line with our findings, the third study found no difference between syndecan-1 levels in COVID-19 patients compared to controls. Although the number of studies was small, the result of our study, which did not find a difference between convalescents and controls, can indicate that glycocalyx damage is more in the active phase of the disease and resolves in COVID-19 survivors. Thus, measuring syndecan-1 as a biomarker is not useful in distinguishing past SARS-CoV-2 infection from patients without a history of COVID-19.

We found no studies evaluating the therapeutic effectiveness of targeting syndecan-1 in COVID-19 patients. However, as CD138 (syndecan-1) is highly expressed in some solid tumors and hematological malignancies [46], studies found new opportunities in treating cancers by targeting syndecan-1 [47]. A preclinical study conducted by Rousseau et al. [48] found that targeting syndecan-1 antigen can be a promising treatment in patients with triple-negative breast cancer. Although antirheumatic medications including methotrexate and tumor necrosis factor (TNF) alpha inhibitors reduced syndecan-1 levels, no studies evaluated the effectiveness of syndecan-1 lowering in COVID-19. Future studies are warranted to evaluate the effect of lowering syndecan-1 levels by specific medications in the disease course of COVID-19.

## Strengths and limitations

The high number of studies evaluating syndecan-1 levels in COVID-19 compared to other biomarkers is the strength of this study. Applying meta-analyses to combine results allowed us to derive more evidence for syndecan-1 as a diagnostic and prognostic biomarker in COVID-19. Finally, following PRISMA guidelines and using independent reviewers in each step of the systematic search was another strength of this study. Although we tried to be flawless and despite being the first meta-analysis study in this field, there are some limitations in this study. First, we were unable to perform meta-analyses to compare different severities of COVID-19 in addition to evaluating syndecan-1 role in predicting ICU admission following SARS-CoV-2 infection. Second, the studies analyzed for COVID-19 vs. healthy controls were among different severities of COVID-19 which we tried to minimize bias using subgroup analysis. Third, some studies not reported exact syndecan-1 levels which prevented us to include them in meta-analyses. Finally, converting median and IQR to mean and SD using methods suggested by Luo et al. [10] and Wan et al. [11] can generate bias.

## Conclusion

In this systematic review and meta-analysis, we found higher levels of syndecan-1 in patients with COVID-19 compared to controls while no difference was found in syndecan-1 levels between COVID-19 convalescents and healthy control subjects. Further studies assessing this marker’s diagnostic and prognostic ability by calculating the sensitivity and specificity of this biomarker are warranted to confirm our findings.

### Electronic supplementary material

Below is the link to the electronic supplementary material.

Supplementary Material 1

## Data Availability

The datasets used and/or analyzed during the current study are available from the corresponding author upon reasonable request.
