# Supplementary material for: The utility of syndecan-1 circulating levels as a biomarker in patients with previous or active COVID-19: a systematic review and meta-analysis
Source: BMC Infect Dis. 2023 Aug 4;23:510. doi: 10.1186/s12879-023-08473-9 (PMC10401738; doi:10.1186/s12879-023-08473-9)
Supplement: Supplementary file 1 — Supplementary Material 1 [file 12879_2023_8473_MOESM1_ESM.docx]

***Supplementary Materials***

***Supplementary Table 1.*** *Search strategy for each database*

| **Query** | | **Results** |
| --- | --- | --- |
| ***PubMed*** | | |
| #1 | ("Syndecan*" OR "CD138" OR "CD-138" OR "CD 138" OR "SDC") | 8,682 |
| #2 | ("COVID-19" OR "coronavirus" OR "SARS-CoV-2" OR "severe acute respiratory syndrome") | 337,651 |
| **#3** | **#1 AND #2** | **77** |
| ***SCOPUS*** | | |
| #1 | (TITLE-ABS-KEY("Syndecan*" OR "CD138" OR "CD-138" OR "CD 138" OR "SDC*")) | 20,559 |
| #2 | (TITLE-ABS-KEY("COVID-19" OR "coronavirus" OR "SARS-CoV-2" OR "severe acute respiratory syndrome")) | 472,840 |
| **#3** | **#1 AND #2** | **116** |
| ***Embase*** | | |
| #1 | (("Syndecan*" OR "CD138" OR "CD-138" OR "CD 138" OR "SDC"):ti,ab,kw) | 13,978 |
| #2 | (("COVID-19" OR "coronavirus" OR "SARS-CoV-2" OR "severe acute respiratory syndrome"):ti,ab,kw) | 351,525 |
| **#3** | **#1 AND #2** | **78** |
| ***Web of Science*** | | |
| #1 | (TS=("Syndecan*" OR "CD138" OR "CD-138" OR "CD 138" OR "SDC")) | 11,502 |
| #2 | (TS=("COVID-19" OR "coronavirus" OR "SARS-CoV-2" OR "severe acute respiratory syndrome")) | 315,915 |
| **#3** | **#1 AND #2** | **49** |
| ***Total*** | | ***320*** |
| ***Total without duplicates*** | | ***201*** |


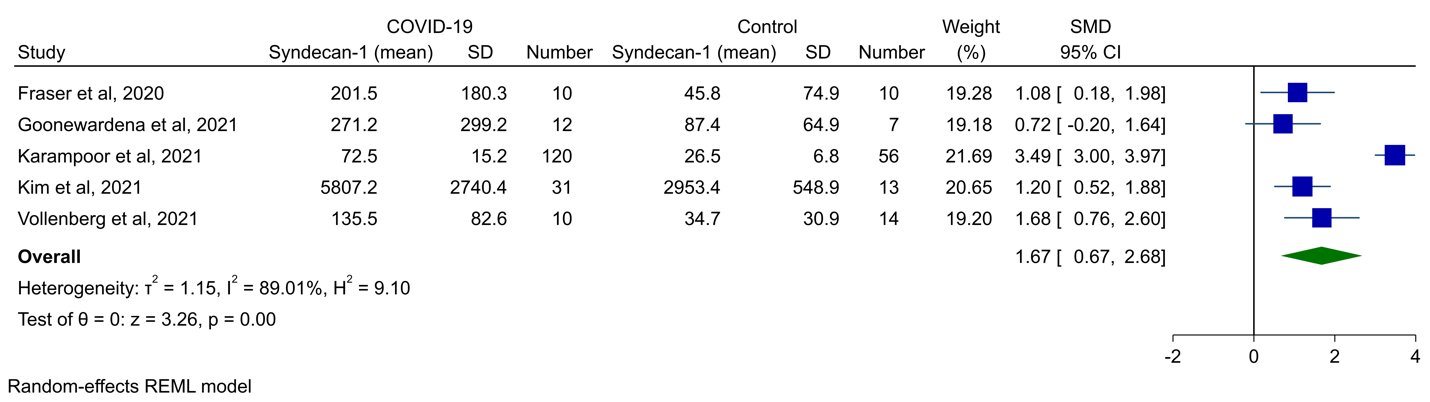


***Supplementary Figure 1.*** *Forest plot for meta-analysis of syndecan-1 levels in comparison of patients with COVID-19 and healthy controls by omitting the study by Mobayen et al. (2021) with hemodialysis population*

***
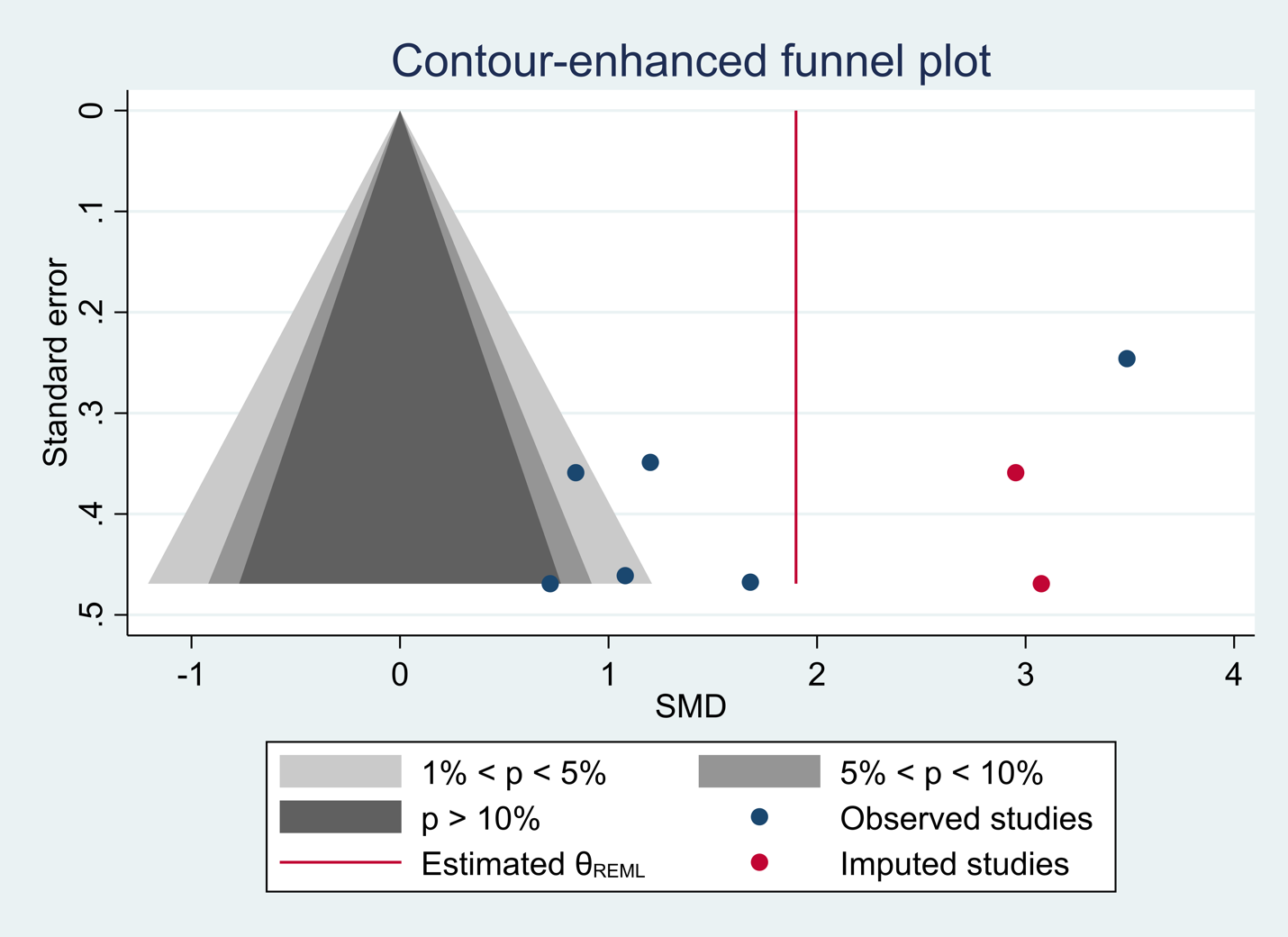
***

***Supplementary Figure 2.*** *Funnel plot for meta-analysis of syndecan-1 levels between COVID-19 patients and healthy controls*

*
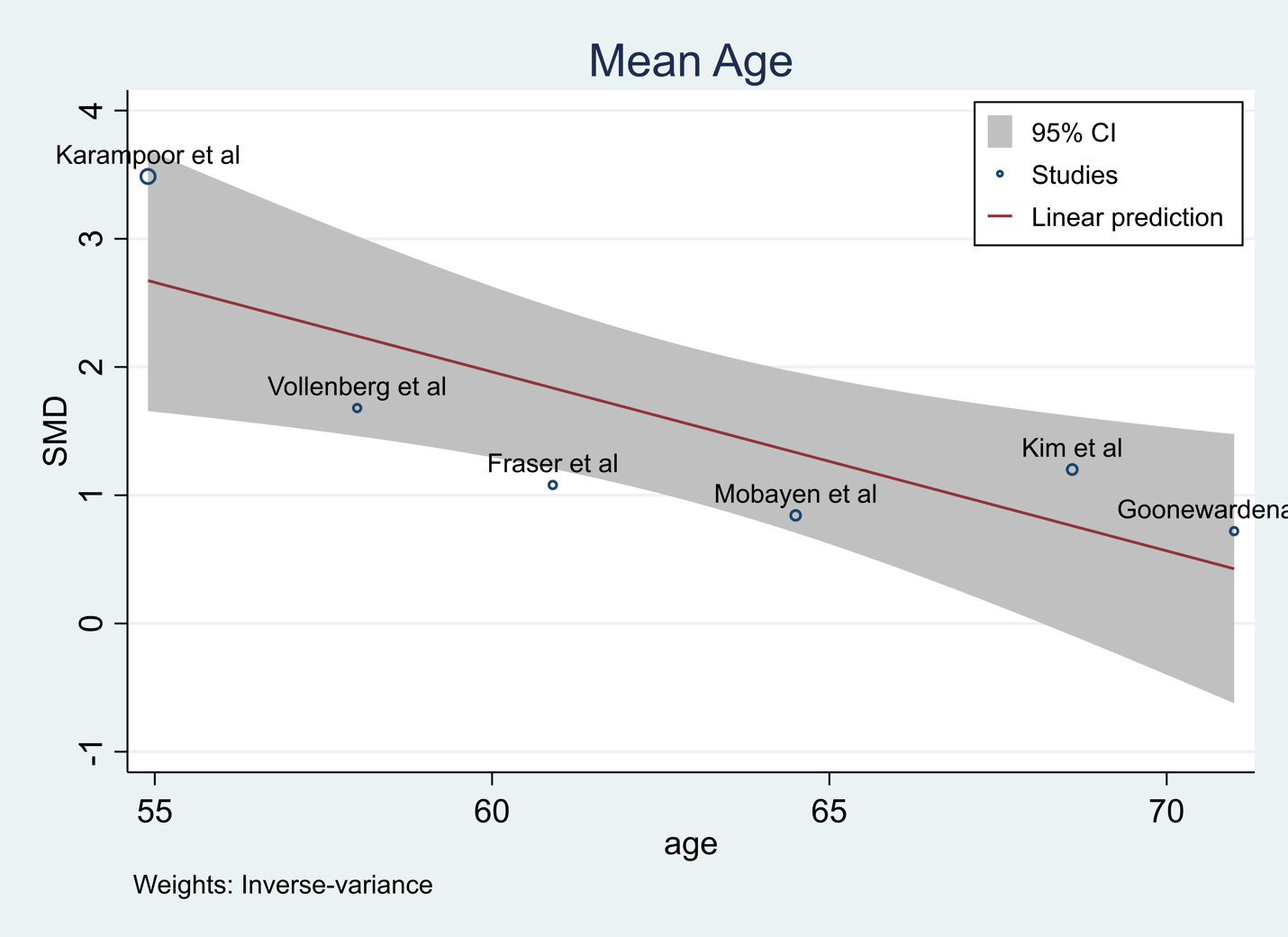
*

***Supplementary Figure 3.*** *Bubble plot showing meta-regression for meta-analysis of syndecan-1 levels between COVID-19 patients and healthy controls based on the mean age of COVID-19 cases*

*
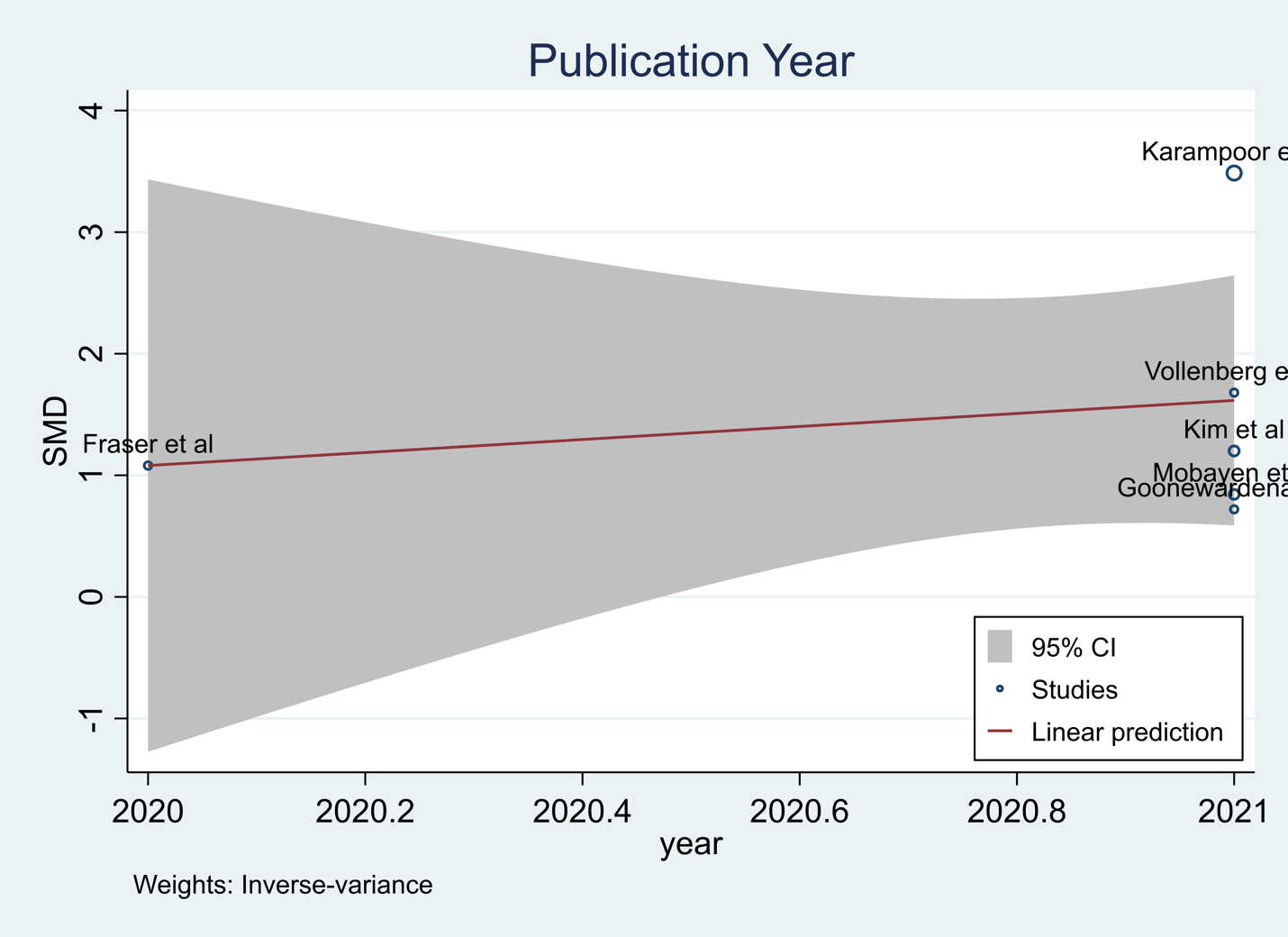
*

***Supplementary Figure 4.*** *Bubble plot showing meta-regression for meta-analysis of syndecan-1 levels between COVID-19 patients and healthy controls based on publication year*

*
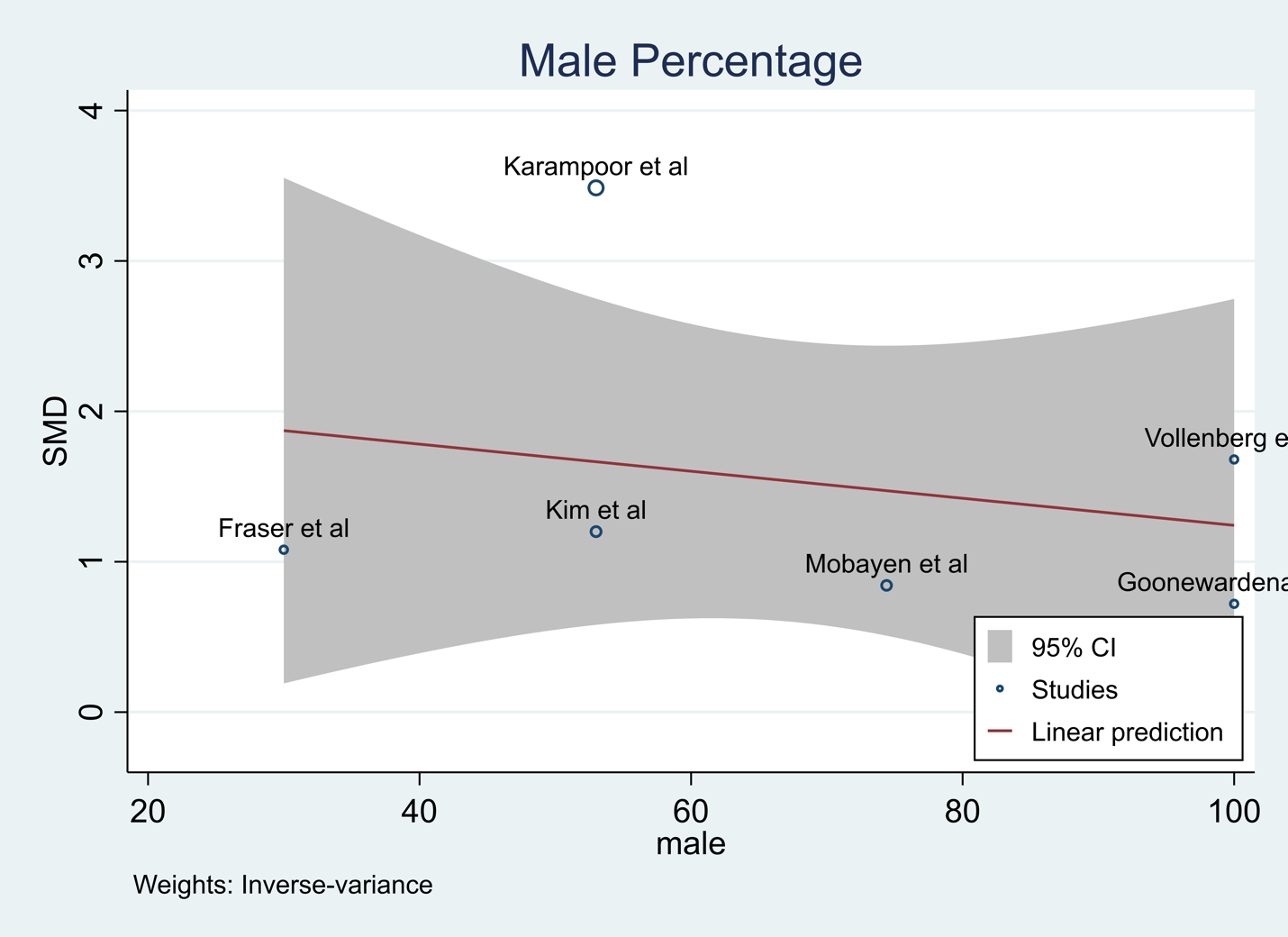
*

***Supplementary Figure 5.*** *Bubble plot showing meta-regression for meta-analysis of syndecan-1 levels between COVID-19 patients and healthy controls based on the male percentage of COVID-19 patients*

*
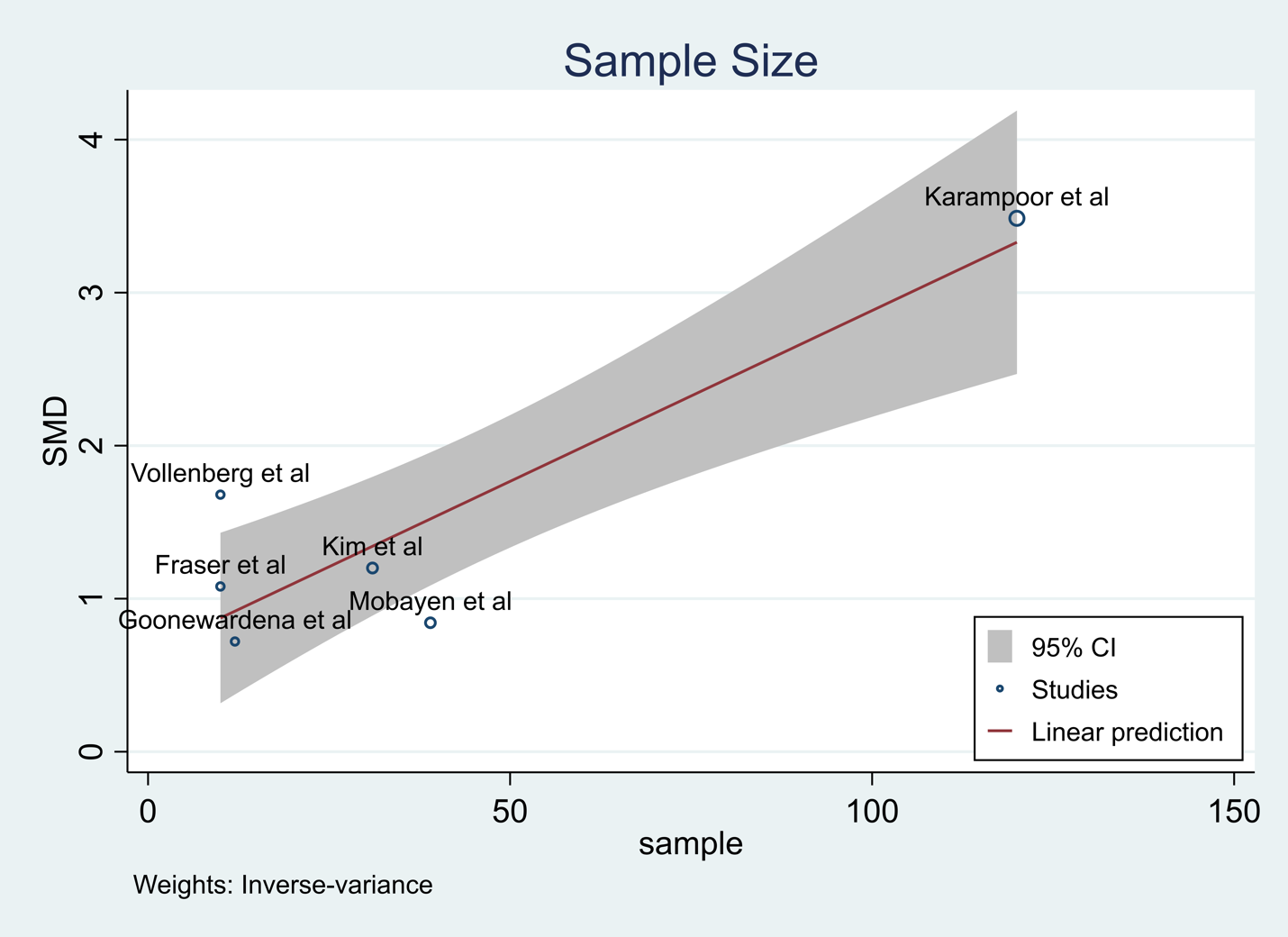
*

***Supplementary Figure 6.*** *Bubble plot showing meta-regression for meta-analysis of syndecan-1 levels between COVID-19 patients and healthy controls based on the sample size of COVID-19 patients*

*
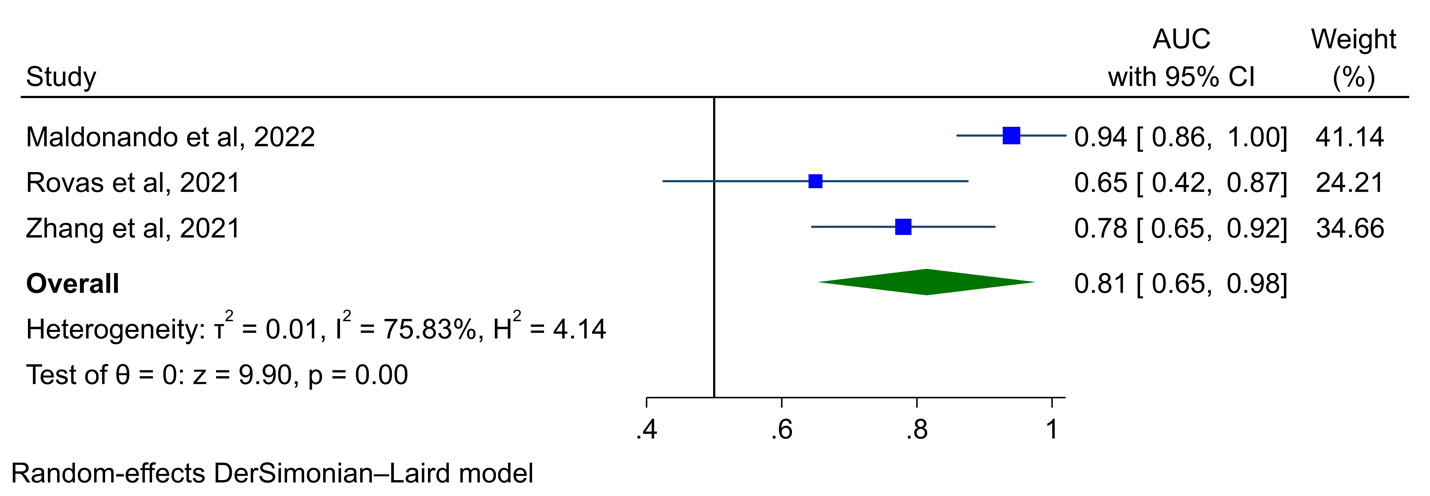
*

***Supplementary Figure 7.*** *Meta-analysis of AUCs predicting COVID-19 mortality in patients using syndecan-1*
